# Dianilinedichloridozinc(II)

**DOI:** 10.1107/S1600536810012274

**Published:** 2010-04-10

**Authors:** Islam Ullah Khan, Ejaz, Onur Şahin, Orhan Büyükgüngör

**Affiliations:** a Materials Chemistry Laboratory, Department of Chemistry, GC University, Lahore 54000, Pakistan; b Department of Physics, Ondokuz Mayıs University, TR-55139 Samsun, Turkey

## Abstract

In the title compound, [ZnCl_2_(C_6_H_7_N)_2_], the Zn^II^ ion (site symmetry 2) adopts a near-regular tetra­hedral ZnN_2_Cl_2_ coordination geometry. In the crystal, mol­ecules are linked by N—H⋯Cl hydrogen bonds, generating (100) sheets containing *R*_2_^2^(8) loops.

## Related literature

For the graph-set analysis of hydrogen-bond patterns, see: Bernstein *et al.* (1995 ▶). For applications of zinc complexes, see: Park *et al.* (2008 ▶) and for a related structure, see: Ejaz *et al.* (2009 ▶).
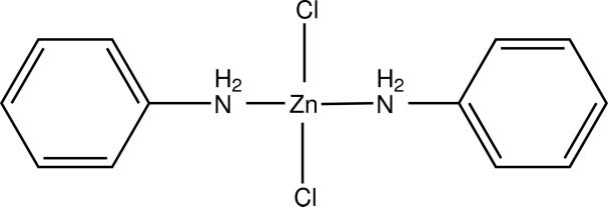


## Experimental

### 

#### Crystal data

[ZnCl_2_(C_6_H_7_N)_2_]
*M* *_r_* = 322.52
Monoclinic, 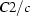

*a* = 26.0713 (7) Å
*b* = 4.7958 (1) Å
*c* = 11.5880 (3) Å
β = 108.823 (1)°
*V* = 1371.39 (6) Å^3^
*Z* = 4
Mo *K*α radiation
μ = 2.16 mm^−1^
*T* = 296 K
0.41 × 0.38 × 0.36 mm

#### Data collection

Bruker Kappa APEXII CCD diffractometer
6369 measured reflections
1687 independent reflections
1523 reflections with *I* > 2σ(*I*)
*R*_int_ = 0.024

#### Refinement

*R*[*F* ^2^ > 2σ(*F* ^2^)] = 0.025
*wR*(*F* ^2^) = 0.102
*S* = 1.01
1687 reflections
86 parameters
2 restraints
H atoms treated by a mixture of independent and constrained refinement
Δρ_max_ = 0.42 e Å^−3^
Δρ_min_ = −0.60 e Å^−3^

### 

Data collection: *APEX2* (Bruker, 2009 ▶); cell refinement: *SAINT* (Bruker, 2009 ▶); data reduction: *SAINT*; program(s) used to solve structure: *SHELXS97* (Sheldrick, 2008 ▶); program(s) used to refine structure: *SHELXL97* (Sheldrick, 2008 ▶); molecular graphics: *ORTEP-3 for Windows* (Farrugia, 1997 ▶); software used to prepare material for publication: *WinGX* (Farrugia, 1999 ▶).

## Supplementary Material

Crystal structure: contains datablocks global, I. DOI: 10.1107/S1600536810012274/hb5384sup1.cif

Structure factors: contains datablocks I. DOI: 10.1107/S1600536810012274/hb5384Isup2.hkl

Additional supplementary materials:  crystallographic information; 3D view; checkCIF report

## Figures and Tables

**Table:** 

Zn1—N1	2.0515 (16)
Zn1—Cl1	2.2454 (5)

**Table:** 

N1—Zn1—Cl1	109.08 (5)

**Table 2:** Hydrogen-bond geometry (Å, °)

*D*—H⋯*A*	*D*—H	H⋯*A*	*D*⋯*A*	*D*—H⋯*A*
N1—H1*A*⋯Cl1^i^	0.85 (2)	2.60 (2)	3.4246 (17)	165 (2)
N1—H1*B*⋯Cl1^ii^	0.86 (2)	2.63 (2)	3.4253 (18)	155 (2)

Symmetry codes: (i) 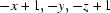; (ii) 
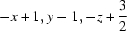
.

**Fig. 1.**
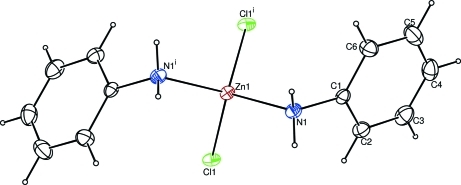
A view of the molecule of (I), showing displacement ellipsoids drawn at the 30% probability level. [Symmetry code: (i) 1-x, y, 3/2-z.]

**Fig. 2.**
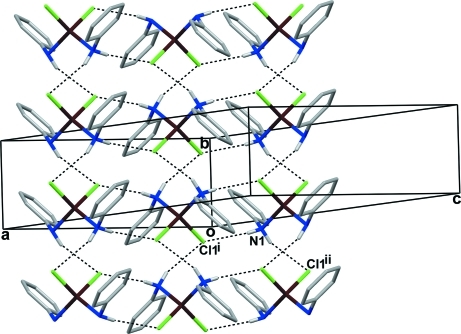
Part of the crystal structure of (I), showing the formation of R22(8) and R43(12) rings. H atoms not involved in these interactions have been omitted for clarity. (Symmetry codes as in Table 2).

## supplementary crystallographic information

### Comment

The title compound is supramolecular complex of Zn^II^ having weak non-classical (N–H···Cl) hydrogen bonds, these non-classical hydrogen bonds act as structural motif for construction of hydrogen bonded polymeric compounds. The intermolecular N–H···Cl hydrogen bond interactions played important role to form a 2-dimensional framework. These hydrogen bonded zinc complexes have shown heterogeneous catalytic activities in some transesterification reaction (Park *et al.*, 2008). The title compound is similar to our previously reported compound ''Dianilinedibromidozinc(II)'' Ejaz *et al.* (2009). Herein, we report the synthesis and crystal structure of the title compound, (I).

The molecular structure of (I) is presented in Fig.1. The compound crystallizes in the space group C2/c with Z'=1/2. The Zn^II^ ion is located on a 2-fold axis and is coordinated by two Cl atoms [Zn1—Cl1/Cl1^i^ = 2.2454 (5) Å] and two amino N atoms from aniline ligands [Zn1—N1/N1^i^ = 2.0515 (16) Å] [symmetry code: (i) 1-x, y, 3/2-z]. The geometry around the Zn^II^ ion is that of a tetrahedral. The benzene ring plane is approximately planar, with maximum deviation from the least-squares plane being 0.005 (2)Å for atom C1.

The amino nitrogen N1 in the molecule at (x, y, z) acts as a hydrogen-bond donor (Table 2) to atom Cl1^i^ so forming a centrosymmetric R_2_^2^(8) (Bernstein *et al.*, 1995) ring centred at (1/2, 0, 1/2). Similarly, amino nitrogen N1 in the molecule at (x, y, z) acts as a hydrogen-bond donor to atom Cl1^ii^ so forming a C(4)[R_2_^2^(8)] chain of rings running parallel to the [0-10] direction. The combination of N—H···Cl hydrogen bonds generates R_4_^3^(12) rings parallel to the bc plane (Fig. 2).

### Experimental

The title compound was synthesized from zinc chloride (0.136 g, 1 mmol) and aniline (0.186 ml, 2 mmol) in methanol (20 ml).  Colourless prisms of (I) were obtained from methanol.

### Refinement

All H atoms bound to C atoms were refined using a riding model, with C—H = 0.93Å and U_iso_(H) = 1.2U_eq_(C) for aromatic C atoms. Amino H atoms were located in difference maps and refined subject to a DFIX restraint of N—H = 0.86 (2) Å.

### Crystal data

**Table:**

[ZnCl_2_(C_6_H_7_N)_2_]	*F*(000) = 656
*M**_r_* = 322.52	*D*_x_ = 1.562 Mg m^−^^3^
Monoclinic, *C*2/*c*	Mo *K*α radiation, λ = 0.71073 Å
Hall symbol: -C 2yc	Cell parameters from 3956 reflections
*a* = 26.0713 (7) Å	θ = 2.9–28.3°
*b* = 4.7958 (1) Å	µ = 2.16 mm^−^^1^
*c* = 11.5880 (3) Å	*T* = 296 K
β = 108.823 (1)°	Prism, colourless
*V* = 1371.39 (6) Å^3^	0.41 × 0.38 × 0.36 mm
*Z* = 4	

### Data collection

**Table:**

Bruker Kappa APEXII CCD diffractometer	1523 reflections with *I* > 2σ(*I*)
Radiation source: fine-focus sealed tube	*R*_int_ = 0.024
graphite	θ_max_ = 28.3°, θ_min_ = 1.7°
phi and ω scans	*h* = −33→34
6369 measured reflections	*k* = −6→6
1687 independent reflections	*l* = −15→15

### Refinement

**Table:**

Refinement on *F*^2^	Primary atom site location: structure-invariant direct methods
Least-squares matrix: full	Secondary atom site location: difference Fourier map
*R*[*F*^2^ > 2σ(*F*^2^)] = 0.025	Hydrogen site location: inferred from neighbouring sites
*wR*(*F*^2^) = 0.102	H atoms treated by a mixture of independent and constrained refinement
*S* = 1.00	*w* = 1/[σ^2^(*F*_o_^2^) + (0.084*P*)^2^] where *P* = (*F*_o_^2^ + 2*F*_c_^2^)/3
1687 reflections	(Δ/σ)_max_ = 0.001
86 parameters	Δρ_max_ = 0.42 e Å^−^^3^
2 restraints	Δρ_min_ = −0.60 e Å^−^^3^

### Special details

**Table:**

Geometry. All esds (except the esd in the dihedral angle between two l.s. planes) are estimated using the full covariance matrix. The cell esds are taken into account individually in the estimation of esds in distances, angles and torsion angles; correlations between esds in cell parameters are only used when they are defined by crystal symmetry. An approximate (isotropic) treatment of cell esds is used for estimating esds involving l.s. planes.
Refinement. Refinement of *F*^2^ against ALL reflections. The weighted *R*-factor wR and goodness of fit *S* are based on *F*^2^, conventional *R*-factors *R* are based on *F*, with *F* set to zero for negative *F*^2^. The threshold expression of *F*^2^ > σ(*F*^2^) is used only for calculating *R*-factors(gt) etc. and is not relevant to the choice of reflections for refinement. *R*-factors based on *F*^2^ are statistically about twice as large as those based on *F*, and *R*- factors based on ALL data will be even larger.

### Fractional atomic coordinates and isotropic or equivalent isotropic displacement parameters (Å^2^)

**Table:**

	*x*	*y*	*z*	*U*_iso_*/*U*_eq_	
C1	0.60906 (8)	−0.0454 (4)	0.73942 (19)	0.0331 (4)	
C2	0.61732 (9)	0.1536 (5)	0.6605 (2)	0.0439 (5)	
H2	0.5901	0.1934	0.5877	0.053*	
C3	0.66631 (11)	0.2925 (5)	0.6906 (3)	0.0576 (6)	
H3	0.6721	0.4253	0.6376	0.069*	
C4	0.70643 (10)	0.2359 (6)	0.7981 (3)	0.0607 (7)	
H4	0.7394	0.3296	0.8179	0.073*	
C5	0.69781 (12)	0.0404 (7)	0.8765 (3)	0.0627 (8)	
H5	0.7250	0.0025	0.9495	0.075*	
C6	0.64892 (10)	−0.1007 (5)	0.8474 (2)	0.0489 (5)	
H6	0.6432	−0.2323	0.9009	0.059*	
N1	0.55735 (7)	−0.1834 (3)	0.70997 (15)	0.0341 (3)	
H1A	0.5471 (11)	−0.230 (6)	0.6350 (17)	0.054 (8)*	
H1B	0.5617 (10)	−0.332 (4)	0.754 (2)	0.045 (6)*	
Cl1	0.45849 (2)	0.32674 (10)	0.58908 (4)	0.04016 (17)	
Zn1	0.5000	0.05523 (6)	0.7500	0.03138 (15)	

### Atomic displacement parameters (Å^2^)

**Table:**

	*U*^11^	*U*^22^	*U*^33^	*U*^12^	*U*^13^	*U*^23^
C1	0.0365 (9)	0.0326 (9)	0.0337 (10)	0.0014 (6)	0.0163 (8)	−0.0056 (7)
C2	0.0493 (11)	0.0451 (11)	0.0397 (11)	−0.0040 (9)	0.0179 (9)	0.0005 (9)
C3	0.0661 (15)	0.0534 (13)	0.0633 (16)	−0.0166 (11)	0.0350 (13)	−0.0052 (11)
C4	0.0435 (12)	0.0645 (15)	0.0768 (18)	−0.0120 (10)	0.0233 (12)	−0.0191 (14)
C5	0.0438 (13)	0.0722 (19)	0.0598 (18)	0.0019 (11)	−0.0002 (12)	−0.0077 (13)
C6	0.0484 (12)	0.0520 (12)	0.0429 (12)	0.0040 (9)	0.0098 (10)	0.0047 (10)
N1	0.0409 (8)	0.0301 (7)	0.0337 (8)	−0.0014 (6)	0.0154 (7)	−0.0032 (6)
Cl1	0.0538 (3)	0.0383 (3)	0.0275 (3)	0.00096 (19)	0.0120 (2)	0.00388 (17)
Zn1	0.0355 (2)	0.0309 (2)	0.0300 (2)	0.000	0.01373 (14)	0.000

### Geometric parameters (Å, °)

**Table:**

C1—C6	1.370 (3)	C5—C6	1.385 (4)
C1—C2	1.386 (3)	C5—H5	0.9300
C1—N1	1.441 (3)	C6—H6	0.9300
C2—C3	1.382 (3)	Zn1—N1	2.0515 (16)
C2—H2	0.9300	N1—H1A	0.852 (17)
C3—C4	1.371 (4)	N1—H1B	0.860 (17)
C3—H3	0.9300	Zn1—Cl1	2.2454 (5)
C4—C5	1.373 (5)	Zn1—N1^i^	2.0515 (16)
C4—H4	0.9300	Zn1—Cl1^i^	2.2454 (5)
			
C6—C1—C2	120.2 (2)	C1—C6—C5	119.6 (3)
C6—C1—N1	120.2 (2)	C1—C6—H6	120.2
C2—C1—N1	119.52 (19)	C5—C6—H6	120.2
C3—C2—C1	119.5 (2)	C1—N1—Zn1	112.63 (11)
C3—C2—H2	120.2	C1—N1—H1A	108.9 (19)
C1—C2—H2	120.2	Zn1—N1—H1A	111.4 (19)
C4—C3—C2	120.4 (3)	C1—N1—H1B	107.8 (16)
C4—C3—H3	119.8	Zn1—N1—H1B	107.1 (17)
C2—C3—H3	119.8	H1A—N1—H1B	109 (3)
C3—C4—C5	119.8 (2)	N1—Zn1—N1^i^	112.17 (9)
C3—C4—H4	120.1	N1—Zn1—Cl1^i^	108.68 (5)
C5—C4—H4	120.1	N1^i^—Zn1—Cl1^i^	109.08 (5)
C4—C5—C6	120.5 (3)	N1—Zn1—Cl1	109.08 (5)
C4—C5—H5	119.8	N1^i^—Zn1—Cl1	108.68 (5)
C6—C5—H5	119.8	Cl1^i^—Zn1—Cl1	109.11 (3)
			
C6—C1—C2—C3	1.0 (3)	C4—C5—C6—C1	0.3 (4)
N1—C1—C2—C3	177.9 (2)	C6—C1—N1—Zn1	97.29 (19)
C1—C2—C3—C4	−0.5 (4)	C2—C1—N1—Zn1	−79.6 (2)
C2—C3—C4—C5	−0.2 (4)	C1—N1—Zn1—N1^i^	−151.65 (16)
C3—C4—C5—C6	0.2 (5)	C1—N1—Zn1—Cl1^i^	−30.96 (15)
C2—C1—C6—C5	−0.9 (4)	C1—N1—Zn1—Cl1	87.90 (14)
N1—C1—C6—C5	−177.8 (2)		

Symmetry codes: (i) −*x*+1, *y*, −*z*+3/2.

### Hydrogen-bond geometry (Å, °)

**Table:**

*D*—H···*A*	*D*—H	H···*A*	*D*···*A*	*D*—H···*A*
N1—H1A···Cl1^ii^	0.85 (2)	2.60 (2)	3.4246 (17)	165 (2)
N1—H1B···Cl1^iii^	0.86 (2)	2.63 (2)	3.4253 (18)	155 (2)

Symmetry codes: (ii) −*x*+1, −*y*, −*z*+1; (iii) −*x*+1, *y*−1, −*z*+3/2.
